# Potential of Cometabolic Transformation of Polysaccharides and Lignin in Lignocellulose by Soil *Actinobacteria*

**DOI:** 10.1371/journal.pone.0089108

**Published:** 2014-02-13

**Authors:** Tomáš Větrovský, Kari Timo Steffen, Petr Baldrian

**Affiliations:** 1 Laboratory of Environmental Microbiology, Institute of Microbiology of the ASCR, v.v.i., Praha, Czech Republic; 2 Department of Applied Chemistry and Microbiology, University of Helsinki, Helsinki, Finland; Oregon State University, United States of America

## Abstract

While it is known that several *Actinobacteria* produce enzymes that decompose polysaccharides or phenolic compounds in dead plant biomass, the occurrence of these traits in the environment remains largely unclear. The aim of this work was to screen isolated actinobacterial strains to explore their ability to produce extracellular enzymes that participate in the degradation of polysaccharides and their ability to cometabolically transform phenolic compounds of various complexities. Actinobacterial strains were isolated from meadow and forest soils and screened for their ability to grow on lignocellulose. The potential to transform ^14^C-labelled phenolic substrates (dehydrogenation polymer (DHP), lignin and catechol) and to produce a range of extracellular, hydrolytic enzymes was investigated in three strains of *Streptomyces* spp. that possessed high lignocellulose degrading activity. Isolated strains showed high variation in their ability to produce cellulose- and hemicellulose-degrading enzymes and were able to mineralise up to 1.1% and to solubilise up to 4% of poplar lignin and to mineralise up to 11.4% and to solubilise up to 64% of catechol, while only minimal mineralisation of DHP was observed. The results confirm the potential importance of *Actinobacteria* in lignocellulose degradation, although it is likely that the decomposition of biopolymers is limited to strains that represent only a minor portion of the entire community, while the range of simple, carbon-containing compounds that serve as sources for actinobacterial growth is relatively wide.

## Introduction

Lignocellulose represents the dominant portion of plant biomass and is thus a key pool of carbon in terrestrial ecosystems. The decomposition of lignocellulose in soil environments, where it originates as aboveground or belowground litter, is thus an essential process of the carbon cycle. Microorganisms represent the key decomposers of lignocellulose in soils and especially fungi are often regarded as major lignocellulose decomposers [1], most likely because their larger, multicellular and often filamentous bodies are better suited for the exploitation of bulky substrates [2]. This potential has led to the evolution of efficient enzymatic systems responsible for the decomposition of biopolymers in several fungi [1], [3]–[5].

The process of lignocellulose decomposition is mediated by extracellular enzymes that target its main components: the polysaccharides cellulose and hemicelluloses and polyphenolic lignin [6]. A wide array of enzymes is necessary for the complete decomposition of lignocellulose. The system for cellulose decomposition typically consists of endocellulases, cellobiohydrolases (exocellulases) and β-glucosidases. The hemicellulolytic system is composed of multiple glycosyl hydrolases that are specific for xylose-, mannose-, arabinose- and galactose-containing polysaccharides; and lignin degradation is mediated by oxidative enzymes, such as oxidases (laccases), peroxidases and auxiliary enzymes, that produce hydrogen peroxide [1], [7], [8].

Although fungi vary largely in their production of extracellular enzymes, several groups, including saprotrophic wood decomposers and cord-forming fungi, that inhabit litter and soil were shown to produce complete arrays of extracellular enzymes that decompose all of the components of lignocellulose [4], [9]. Current advances in genome sequencing indicate that the theoretical potential of bacteria to degrade certain components of lignocellulose, e.g., cellulose, is relatively widespread [10]; and, for certain taxa, enzymes involved in decomposition were characterised [11]. Moreover, recent reports also show that bacteria may play a significant role in cellulose decomposition in soil environments [12]. However, the composition of bacterial enzymatic systems has not been systematically addressed, and it is difficult to estimate their potential to transform individual lignocellulose components.

*Actinobacteria* seem to be good candidates for efficient lignocellulose decomposition, and their filamentous growth may help them access and utilise polymeric substrates [13]. Therefore, the involvement of certain *Actinobacteria* in the degradation of polysaccharides or phenolic compounds in dead plant biomass is generally accepted [14], [15]. This is based on previous reports that suggest the presence of decomposer traits in several actinobacterial taxa. In the case of cellulose, the production of endocellulase by the genera *Streptomyces*, *Cellulomonas* and *Acidothermus* was reported [16]–[18], while efficient exocellulases often combined with xylanase activity were found in *Thermobifida*, *Cellulomonas* and *Cellulosimicrobium* [19]–[21]. β-Glucosidases have been characterised in the above genera as well as in *Clavibacter*, *Terrabacter*, *Micrococcus*, *Microbacterium* and *Bifidobacterium* [22]–[26]. Recently, many putative cellulose-degrading enzymes were found in the sequenced genomes of several *Actinobacteria* [27], and this phylum showed the highest percentage of genomes that harboured putative cellulolytic enzymes. Approximately 1/3 of the 514 characterised genomes harboured at least one putative cellulase [10]. Although information on hemicellulose-degrading enzymes is scarce, individual enzymes were reported in multiple genera, including *Streptomyces*, *Cellulomonas*, *Cellulosimicrobium* and *Kocuria* [28]–[31].

Although the major lignin degraders are white-rot fungi, there are also many reports about bacterial strains that are able to degrade lignin. In addition to the *Proteobacteria* and *Firmicutes* [32]–[34], these reports also mention actinobacterial taxa. Studies on the decomposition of natural and synthetic lignins, for example, isolated lignin, prepared ^14^C-synthetic lignins or model compounds, indicated that the genera *Arthrobacter*, *Nocardia* and *Streptomyces* were capable of lignin utilisation, although their efficiencies varied widely and did not reach the level that was observed in ligninolytic fungi [35]–[38].

Despite the relative abundance of reports on the decomposer abilities of *Actinobacteria*, the current information remains rather fragmented. Different strains have been studied for the production of individual enzymes, and the abilities to use plant lignocellulose as a growth substrate were not studied in much detail. The aim of this study was to explore the ability of soil *Actinobacteria* to act as decomposers of dead plant biomass. To achieve this goal, a set of natural isolates was screened for their production of cellulolytic enzymes, and active strains were tested for their ability to use lignocellulose in the form of wheat straw as a growth source. The potential decomposers were further screened for the production of multiple lignocellulose-degrading enzymes and for their ability to cometabolically transform ^14^C-labelled phenolic substances (lignin, DHP and catechol) during their growth on wheat straw. The *Actinobacteria* in this study were isolated from soil with mixed-metal pollution. Due to their heavy metal resistance, *Actinobacteria* are frequently found in such soils, and this group may replace the more sensitive fungi as the main decomposers in such soils [39], [40]. We thus expected that the decomposition of lignocellulose would be a common trait of strains isolated from this environment.

## Materials and Methods

### Isolation of Bacterial Strains

The study was carried out on private land. No specific permission was required for the activities covered by this study. However, for more intensive research activities, individual private owners have to grant the permit to conduct such research. The field studies did not involve endangered or protected species. Bacterial strains were isolated from the organic horizons of the grassland and forest soils near Příbram, Czech Republic (49°42′22.207″N, 13°58′27.296″E). The soil is a cambisol with pH of approximately 5.5 and has a clay/silt/sand ratio of approx. 40∶30:30% and an elevated heavy metal content (namely, Cu, Zn, Cd, Zn, Pb and As) due to its location near a polymetallic smelter [41]. The study was performed on private land; no specific permit was required for the activities performed as part of this study.

Physical and chemical treatments were used for the selective isolation of soil *Actinobacteria*. Soil samples were pre-treated by dry heating (120°C) and phenol treatment (1.5%), and water extracts were used to inoculate plates containing selective media, either humic acid-vitamin agar (1 g L^−1^ humic acid, 0.5 g L^−1^ Na_2_HPO_4_, 7.7 g L^−1^ KCl, 0.05 g L^−1^ MgSO_4_.7H_2_O, 0.01 g L^−1^ FeSO_4_.7H_2_O, 0.02 g L^−1^ CaCO_3_, B-vitamins: 0.5 mg L^−1^ thiamine-HCl, riboflavin, niacin, pyridoxine, inositol, Ca-pantothenate, *p*-aminobenzoic acid and 0.25 mg L^−1^ biotin, 18 g L^−1^ agar, pH 7.2) or lignin-soy bean flour-vitamin agar containing soil extract (1 g L^−1^ lignin, 0.2 g L^−1^ soy bean flour, 0.5 g L^−1^ Na_2_HPO_4_, 7.7 g L^−1^ KCl, 0.05 g L^−1^, MgSO_4_.7H_2_O, 0.01 g L^−1^ FeSO_4_.7H_2_O, 0.02 g L^−1^ CaCO_3_, B-vitamins (see above), 100 mL L^−1^ soil extract, 18 g L^−1^ agar, pH 7.5) that was supplemented with the antibiotics kanamycin (20 mg L^−1^) and nalidic acid (10 mg L^−1^) [42], [43]. Pure cultures of bacteria were obtained from agar plates, and those strains that were identified as *Actinobacteria* were retained. Strains were stored in a sterile, 50% glycerol solution in 25 mM Tris at −20°C and subcultured on GYM agar (4 g L^−1^ glucose, 4 g L^−1^ yeast extract, 10 g L^−1^ malt extract, 2 g L^−1^ CaCO_3_, 12 g L^−1^ agar, pH 7.2) at 25°C.

### Screening for Lignocellulose-degrading Strains

Efficient decomposition of cellulose, which is the major component of dead plant biomass, depends on the production of cellobiohydrolase and β-glucosidase. To screen for the production of these two enzymes, isolated actinobacterial strains were cultivated in liquid GYM medium for 14 days at 25°C without agitation (three replicates). The cultivation liquid was collected, and the activities of the extracellular enzymes were measured spectrophotometrically as described previously [44]. Cellobiohydrolase (exocellulase, EC 3.2.1.91) and 1,4-β-glucosidase (EC 3.2.1.21) were assayed using 4-methylumbelliferyl-β-D-cellobioside and 4-methylumbelliferyl-β-D-glucopyranoside, respectively, in 50 mM sodium acetate buffer (pH 5.0). The reaction mixtures were incubated at 40°C for 120 min and terminated by sodium carbonate addition [44].

The activity of each strain was ranked on a scale of negative, low, medium or high. Cellobiohydrolase: negative = 0−0.5 µmol min^−1^ mL^−1^, low = from >1 to 15 µmol min^−1^ mL^−1^, medium from >15 to 50 µmol min^−1^ mL^−1^, and high >50 µmol min^−1^ mL^−1^. 1,4-β-glucosidase: negative = 0–1 µmol min^−1^ mL^−1^, low = from >1 to 50, medium from >50 to 200 µmol min^−1^ mL^−1^, and high >200 µmol min^−1^ mL^−1^. Strains that produced cellobiohydrolase and 1,4-β-glucosidase and exhibited high activity of at least one of those enzymes were identified, and their ability to grow on lignocellulose as a carbon source was examined. One gram of air-dried, milled wheat straw was added into 100-mL, thick-walled flasks to form a uniform layer. Each flask was supplemented with 5 mL of distilled water and sterilised by autoclaving (2×30 min at 121°C with cooling to room temperature between the two cycles). The flasks were inoculated with 1 mL of cell suspension that had been pre-grown for three days on liquid GYM media. Triplicate flasks for each strain were incubated for 21 days at 25°C. After incubation, the enzymes were extracted in 15 mL of distilled water, and the extracts were filtered and used for enzyme activity measurements.

The activities of 1,4-β-glucosidase, cellobiohydrolase and 1,4-β-xylosidase in the extracts were assessed using 4-methylumbelliferyl-β-D-glucopyranoside, MUF-β-D-cellobioside and MUF-β-D-xylopyranoside, respectively, in 50 mM sodium acetate buffer, pH 5.0, as previously described [44]. Substrates (100 µL in DMSO) at a final concentration of 500 µM were combined with the three technical replicates of the 100-µL extracts in a 96-well multiwell plate. For the background fluorescence measurement, 100 µL of sodium acetate buffer was combined with 100 µL of the 4-methylumbelliferol standards to correct for fluorescence quenching. The multiwell plates were incubated at 40°C, and fluorescence was recorded from 5 min to 125 min using the Infinite microplate reader (TECAN, Austria) at an excitation wavelength of 355 nm and an emission wavelength of 460 nm. The quantitative enzymatic activities after blank subtraction were calculated based on standard curves of 4-methylumbelliferone, and enzyme activity was expressed per g of straw dry mass.

Of the 14 strains, only six exhibited visual growth and produced extracellular enzymes in straw. Among these, three strains, pl88, pr6 and pr55, that highly produced cellobiohydrolase were selected for the detailed characterisation of glycosyl hydrolase production and the decomposition of phenolic compounds.

To analyse the spectra of the extracellular enzymes that were produced by the bacterial strains, pl88, pr6 and pr55 were cultivated on diluted GYM media with either cellulose as a specific inducer or with finely milled wheat straw (mesh size 0.2 mm) as a complex inducer. In 50-mL flasks, 10 mL of 10× diluted GYM media was combined with 50 mg of cellulose or wheat straw and sterilised by autoclaving. The flasks were inoculated with 100 µL of cell suspension that had been pre-grown for three days in liquid GYM media. Triplicate flasks for each strain were incubated for 21 days at 25°C. After incubation, the 1,4-β-glucosidase, cellobiohydrolase and 1,4-β-xylosidase activities were measured, as described above. The activities of 1,4-α-glucosidase, 1,4-α-arabinosidase, 1,4-β-galactosidase, 1,4-β-mannosidase and 1,4-β-glucuronidase in the extracts were assessed using 4-methylumbelliferyl-α-D-glucopyranoside, 4-methylumbelliferyl-α-L-arabinopyranoside, 4-methylumbelliferyl-β-D-galactopyranoside, 4-methylumbelliferyl-β-D-mannopyranoside and 4-methylumbelliferyl-β-D-glucuronide, respectively, and the same method. The activities of endo-1,4-β-glucanase (endocellulase) and endo-1,4-β-xylanase (endoxylanase) were assayed using azo-dyed carboxymethyl cellulose and birchwood xylan, respectively, according to the manufacturer’s instructions (Megazyme, Ireland). Reaction mixture containing 0.2 mL of a 2% dyed substrate in 200 mM sodium acetate buffer, pH 5.0, and 0.2 mL of sample was incubated at 40°C for 60 min, and the reaction was ended by adding 1 mL of ethanol followed by 10 s of vortexing and 10 min of centrifugation (10,000×g) [45]. The amount of released dye was measured at 595 nm, and the enzyme activity was calculated according to standard curves that correlated dye release with the release of reducing sugars.

### Transformation of ^14^C-labelled Phenolic Compounds

The transformation of phenolic compounds of various complexities was studied using ^14^C-labelled compounds. ^14^C_β_-labelled dehydrogenation polymer (^14^C-DHP) was synthesised according to Brunow [46] and dissolved in a N,N-dimethylformamide-water suspension (1∶20 v/v) [47]. ^14^C_β_-labelled lignin was extracted from labelled poplar trees that were prepared according to Odier [48] and used as a solid material. ^14^C_β_-labelled catechol in an ethanol solution (Sigma) that was mixed with water (1∶18.75 v/v) was used directly.

The cometabolic transformation of phenolic compounds was studied in 100-mL, thick-walled flasks containing 1 g of air-dried and milled wheat straw, which was added to form a uniform layer. Each flask was supplemented with 5 mL of distilled water and sterilised by autoclaving (2×30 min at 121°C with cooling to room temperature between the two cycles). The flasks were inoculated with 1 mL of cell suspension that had been pre-grown for three days in liquid GYM media (five flasks per strain). Control flasks were left uninoculated. The following day, ^14^C-labelled DHP, catechol and poplar lignin were added. In the DHP flasks, 750 µL of^ 14^C-DHP in a N,N-dimethylformamide-water suspension was added drop-wise onto the surface of the straw layer, which resulted in a final radioactivity of 127,500 dpm per flask. In the catechol flasks, 750 µL of ^14^C-catechol in an ethanol-water solution was added, which resulted in a final radioactivity of 550,500 dpm per flask. In the lignin flasks, 10 mg of fine, ^14^C-poplar lignin powder was added onto the surface of the straw layer, which resulted in a final radioactivity of 230,000 dpm per flask, and then, 750 µL of sterile water was added. The flasks were sealed with rubber septa and aluminium caps.

Incubation proceeded for 76 days at 24°C in the dark. Volatile compounds were flushed out of the flasks every week using sterile air, and CO_2_ was trapped by bubbling the released air through two sequential flasks containing Opti-Fluor and Carbosorb/Opti-Fluor (Packard Instruments) every week. A liquid scintillation counter (Wallac 1411, WallacOy, Finland) was used to quantify the trapped ^14^CO_2_ during the experiment. After incubation, the flasks supplemented with the ^14^C-labelled substances were stored at −18°C until mass-balance extraction.

The flasks containing the residual ^14^C material were extracted twice with 6 mL of distilled water. After the addition of water, the incubation flasks were shaken for 2 h (280 rpm) at room temperature on a table rotary shaker. The suspension was then poured into a 60-mL syringe containing a pre-weighed cotton plug, and the aqueous extract was pushed through the syringe. For measurements, 1 mL of water extract was diluted with 7 mL of distilled water, and the dilutions were mixed with 10 mL of LUMAGEL. The radioactivity of each extract was measured using a liquid scintillation counter (Model 1411, WallacOy, Finland). The cotton plugs that were used in the filtrations and the residual solid material were air dried and then burned in a combustion chamber (Junitek, Finland). The radioactivity was then counted using a liquid scintillation counter as reported previously [49]. The efficiency of combustion was verified using ^14^C-labelled standards.

A one-way analysis of variance with the Fisher’s least significant difference *post hoc* test was used to analyse the statistical significance of differences among treatments. Differences with a P<0.05 were regarded as statistically significant.

### Identification of Actinobacterial Strains

DNA was isolated from the actinobacterial biomass that was obtained by cultivation in liquid GYM medium using the modified Miller-SK method [50]. Isolated genomic DNA was used as a template in PCR reactions using universal primers for the bacterial and archaeal 16 S rRNA gene, pH-T7 (5‘-TAATACGACTCACTATAGAGAGTTTGATCCTGGCTCAG-3‘) and pA (5‘-AAGGAGGTGATCCAGCCGCA-3‘). Each 50-µl reaction mixture contained 5 µl of 10× buffer for DyNAzyme DNA Polymerase (Finnzymes), 3 µl of purified BSA (10 mg mL^−1^), 2 µl of each primer (0.01 mM), 1 µl of PCR Nucleotide Mix (10 mM each), 1.5 µl of DyNAZyme II DNA Polymerase (2 U µl^−1^, Finnzymes) and 1 µl of isolated genomic DNA. The cycling conditions were as follows: 1× 94°C 5 min, 35× (94°C 1 min, 57°C for 45 s min and 72°C for 90 s) followed by 72°C for 10 min. The PCR products were directly sequenced by Macrogen (Seoul, Korea), and the sequences were manually edited using the BioEdit program (http://www.mbio.ncsu.edu/bioedit/bioedit.html) and corrected prior to a BLASTn search against the nucleotide database at the NCBI (http://www.ncbi.nlm.nih.gov/blast).

## Results and Discussion

Seventy-six strains of soil *Actinobacteria* were isolated from the soils of the study area and screened for their ability to produce enzymes involved in cellulose decomposition, including cellobiohydrolase and 1,4-β-glucosidase. Of these strains, 32% did not produce any of the tested enzymes, while 31% produced both of them. The production of 1,4-β-glucosidase was more common (57% of strains) than that of cellobiohydrolase (41% of strains; Table 1). The percentage of strains that produced cellobiohydrolase roughly corresponded to the percentage of actinobacterial strains that harboured a gene for endocellulase or cellobiohydrolase (i.e., the glycosyl hydrolase family GH5, 6, 8, 9, 12, 44, 45 or 48), which was one-third of all of the sequenced actinobacterial genomes that were analysed in a recent study [10]. The percentage of strains that did not produce detectable amounts of any enzyme (32%) was higher than what was inferred from the analysis of the genomes, which was less than 20% of the genomes [10]. It is thus possible that some of the strains that harbour 1,4-β-glucosidase do not express the gene or show only low expression levels.

**Table 1:** Screening of actinobacterial strains for their ability to produce cellobiohydrolase and 1,4-β-glucosidase.

Strain	Cellobiohydrolase	1,4-β-Glucosidase
pl18	++	+++
pl21	−	+
pl23	+	++
pl28	+	++
pl36	+++	+++
pl41	+++	+++
pl67	++	−
pl70	−	++
pl73	−	+
pl75	−	+
pl77	−	+++
pl80	−	+
pl81	−	+
pl84	+	++
pl86	+	−
pl88	+++	+++
pl95	+++	+++
pl98	−	+
pl100	−	+
pl107	−	++
pl112	−	+
pl116	+++	+++
pl118	+	+++
pl123	+++	+++
pl124	++	+
pl129	+	+
pl131	−	++
pl134	−	+++
pl136	−	++
pl138	−	+++
pl149	+	++
pl150	−	+++
pl153	+++	+++
pl154	++	+++
pr10	+	−
pr22	−	+++
pr24	+	−
pr3	+	+
pr30	+	+++
pr4	−	+
pr40	++	−
pr41	+++	++
pr45	−	+++
pr48	−	++
pr49	+	+
pr52	++	−
pr55	+++	+
pr57	+	++
pr6	+++	++
pr7	+	−
pr9	++	−

Twenty-five isolates produced neither of the enzymes. Legend: “+++” high production of enzyme (activity >100 mU/mL), “++” average production (100> activity >10), small production (activity <10) and “–” no enzyme production.

**Figure 1:**
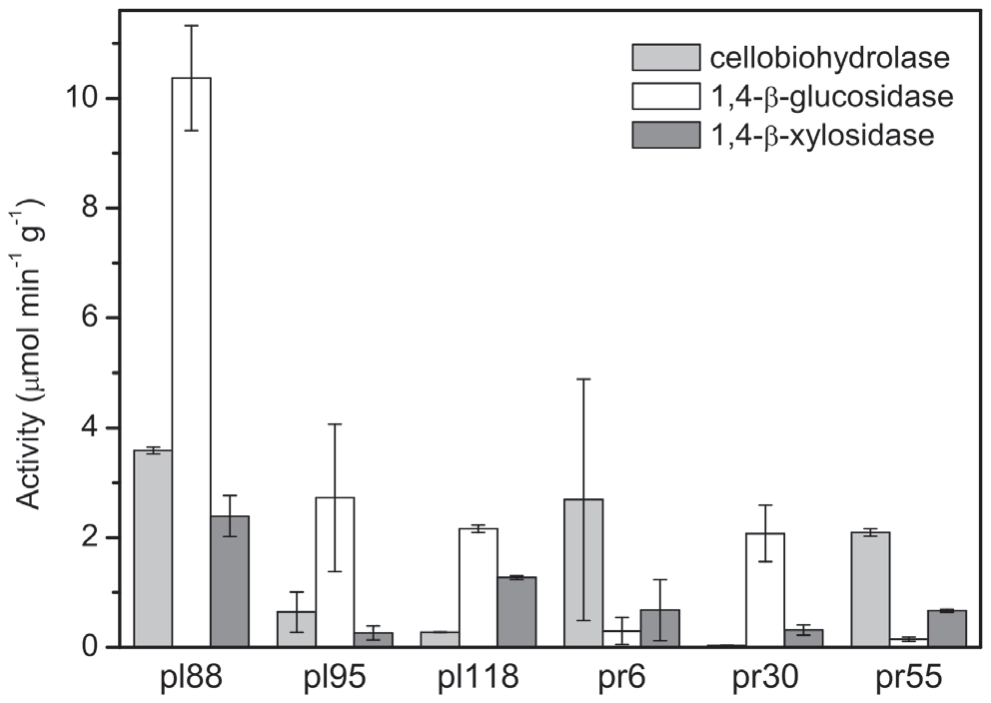
Production of cellobiohydrolase, β-glucosidase and β-xylosidase by *Actinobacteria*. Activity of cellobiohydrolase, 1,4-β-glucosidase and 1,4-β-xylosidase after a 21-day cultivation of the selected actinobacterial strains on wheat straw. The data represent the means and standard errors.

Fourteen strains that produced both enzymes, and highly produced at least one, were selected for further studies and were identified by 16S rRNA sequencing. Of these, eight strains showed highest similarity with members of the genus *Streptomyces,* while the best hits for the others were from the genera *Amycolatopsis*, *Curtobacterium*, *Kribbella*, *Microbispora*, *Micromonospora* and *Nocardia* (Table 2). The activity of cellobiohydrolase and 1,4-β-glucosidase in the genera *Amycolatopsis*, *Kribella*, *Micromonospora*, *Nocardia* and *Streptomyces* corresponded well with the presence of the corresponding genes in their genomes [10]. The currently analysed genomes of *Nocardia* did not contain a cellobiohydrolase gene, and the genomes of *Curtobacterium* and *Microbispora* have not been analysed. Despite their high cellulolytic activity, only six of the fourteen analysed isolates (pl88, pl95, pl118, pr6, pr30 and pr55) showed visually detectable growth on milled wheat straw after 21 days of culturing. All of these strains produced extracellular glycosyl hydrolases: cellobiohydrolase, 1,4-β-glucosidase and 1,4-β-xylosidase, although their activities differed (Figure 1). The strains that highly produced cellobiohydrolase (>2 µmol min^−1^ g^−1^ straw dry mass) were selected for further experiments. Cellobiohydrolase represents the rate-limiting enzyme in the decomposition of cellulose, the most abundant and rapidly decomposable polysaccharide in plant litter [12], [51].

**Table 2:** Identification of selected actinobacterial strains and the accession numbers of their partial 16S rRNA gene sequences.

Strain	Accession No	Closest hit	Accession No	Similarity (%)	Coverage (%)
pl18	KC789721	*Micromonospora saelicesensis* strain L6	JN862845	99.6	99.3
pl36	KC789723	*Streptomyces ciscaucasicus* strain HBUM83169	EU841585	99.9	99.8
pl41	KC789724	*Curtobacterium flaccumfaciens* strain LMG 3645	NR025467	100.0	99.3
pl88	KC789730	*Streptomyces atratus* strain HBUM173340	FJ486302	100.0	94.2
pl95	KC789731	*Streptomyces aureus* strain HBUM174596	EU841581	99.4	100.0
pl116	KC789734	*Kribbella antibiotica* strain YIM 31530	NR029048	98.8	99.8
pl118	KC789719	*Streptomyces sanglieri* strain IHB B 6004	KF475877	99.6	99.9
pl123	KC789735	*Nocardia exalbida* W9709	GQ376167	99.2	100.0
pl153	KC789738	*Streptomyces hygroscopicus* subsp. *geldanus* strain NBRC14620	AB184606	99.8	99.9
pl154	KC789739	*Microbispora rosea* subsp. *rosea* strain A011	AB369120	99.8	95.6
pr6	KC789740	*Streptomyces mauvecolor* strain 7534	JN180187	99.2	99.3
pr30	KC789741	*Amycolatopsis saalfeldensis* strain HKI 0474	DQ792502	99.2	100.0
pr41	KC789742	*Streptomyces setonensis* strain 17-1	EU367980	99.4	99.1
pr55	KC789744	*Streptomyces sannanensis* strain 126195	JN180213	98.9	99.4

**Figure 2:**
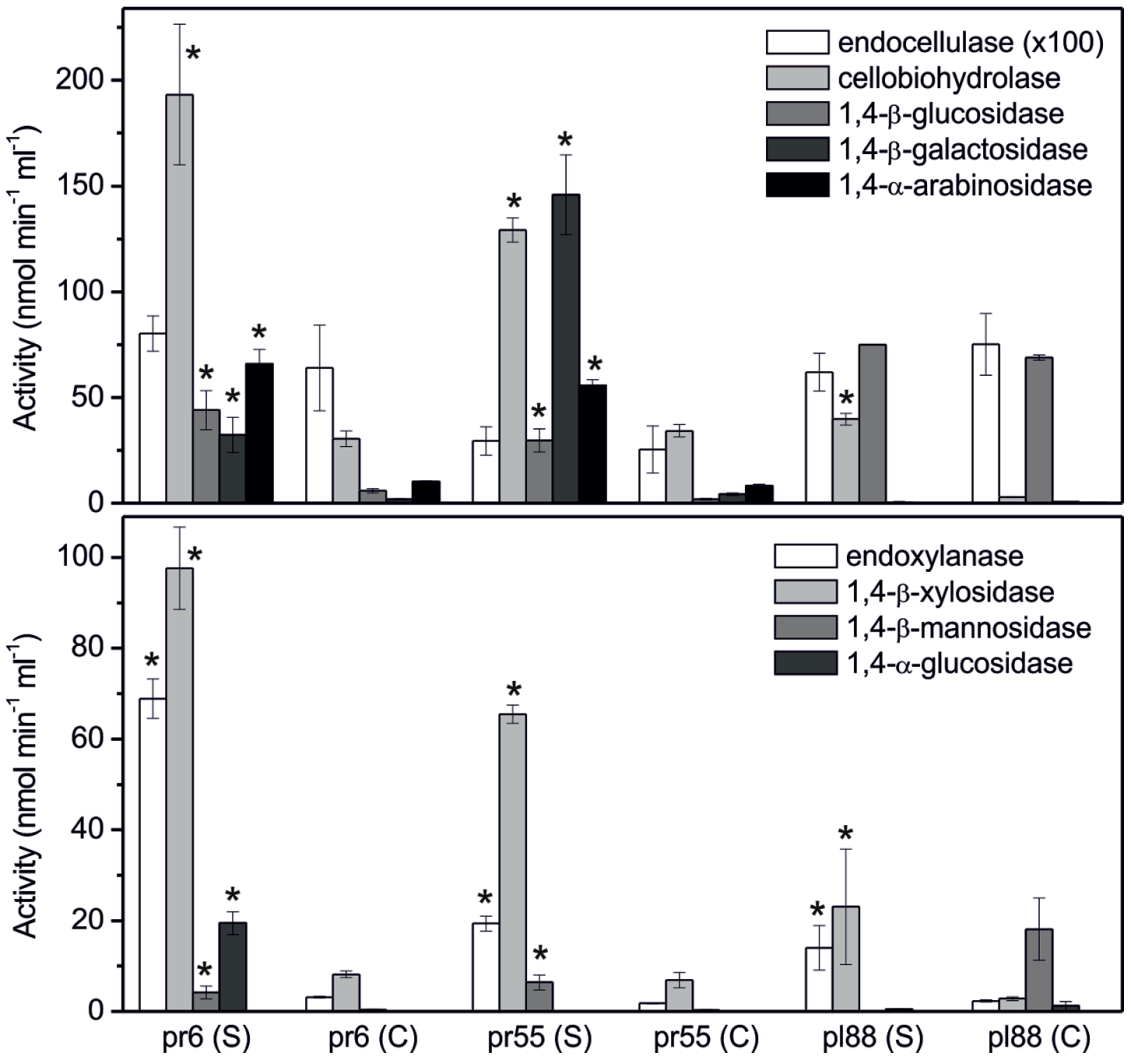
Production of hydrolytic enzymes by selected *Actinobacteria*. Activity of glycosyl hydrolases after a 21-day cultivation of the selected actinobacterial strains on wheat straw (S) and cellulose (C). The data represent the means and standard errors. The activity of endocellulase was multiplied 100× to fit the same scale. Asterisks indicate significant difference (P<0.05) in enzyme activity among treatments.

All studied strains produced a complete set of cellulolytic enzymes: endocellulase, cellobiohydrolase and 1,4-β-glucosidase, and all strains produced the xylanolytic enzymes endoxylanase and 1,4-β-xylosidase as well as the hemicellulases 1,4-β-galactosidase and 1,4-β-mannosidase. 1,4-α-Arabinosidase was only produced by pr6 and pr55, while amylase was produced by pr6 and pl88. The bacteria produced the same enzymes, regardless of whether cellulose or milled wheat straw was used as the carbon source (Figure 2). However, wheat straw, which is a complex substrate that contains various polysaccharides, increased the production of most hemicellulases and cellobiohydrolase and, in the case of pr6 and pr55, the production of 1,4-β-glucosidase. The titres of exocellulase were similar in both treatments. Remarkably, the production of endocellulase was 10–100× lower than that of endoxylanase, and the activities of 1,4-β-xylosidase on straw were higher than that of 1,4-β-glucosidase. Unfortunately, the production of xylanase was not studied in *Actinobacteria* in sufficient detail; however, studies on environmental isolates indicated that this ability might be relatively common [52]. When the genes of the most common endoxylanase, GH10, were screened across a range of different soils, actinobacterial genes were recovered with a relatively high frequency [53]. The activity of 1,4-β-arabinosidase was higher than that of 1,4-β-glucosidase in the pr6 and pr55 strains. It is thus possible that hemicellulose, and particularly xylan, represents a preferred source for lignocellulose-degrading *Actinobacteria*. Although cellulose decomposition was found to be more rapid in decomposing plant litter than that of hemicelluloses, the utilisation of xylose and arabinose-containing hemicelluloses was relatively fast [51]. Although the production of 1,4-β-mannosidase, 1,4-β-galactosidase and 1,4-α-arabinosidase by individual taxa of *Actinobacteria* was reported previously [54]–[56], we show that they belong to a set of enzymes that are simultaneously produced by saprotrophic taxa during their growth on lignocellulose. The enzyme 1,4-β-glucuronidase, which is involved in the degradation of pectins, was not produced by the tested strains.

**Figure 3:**
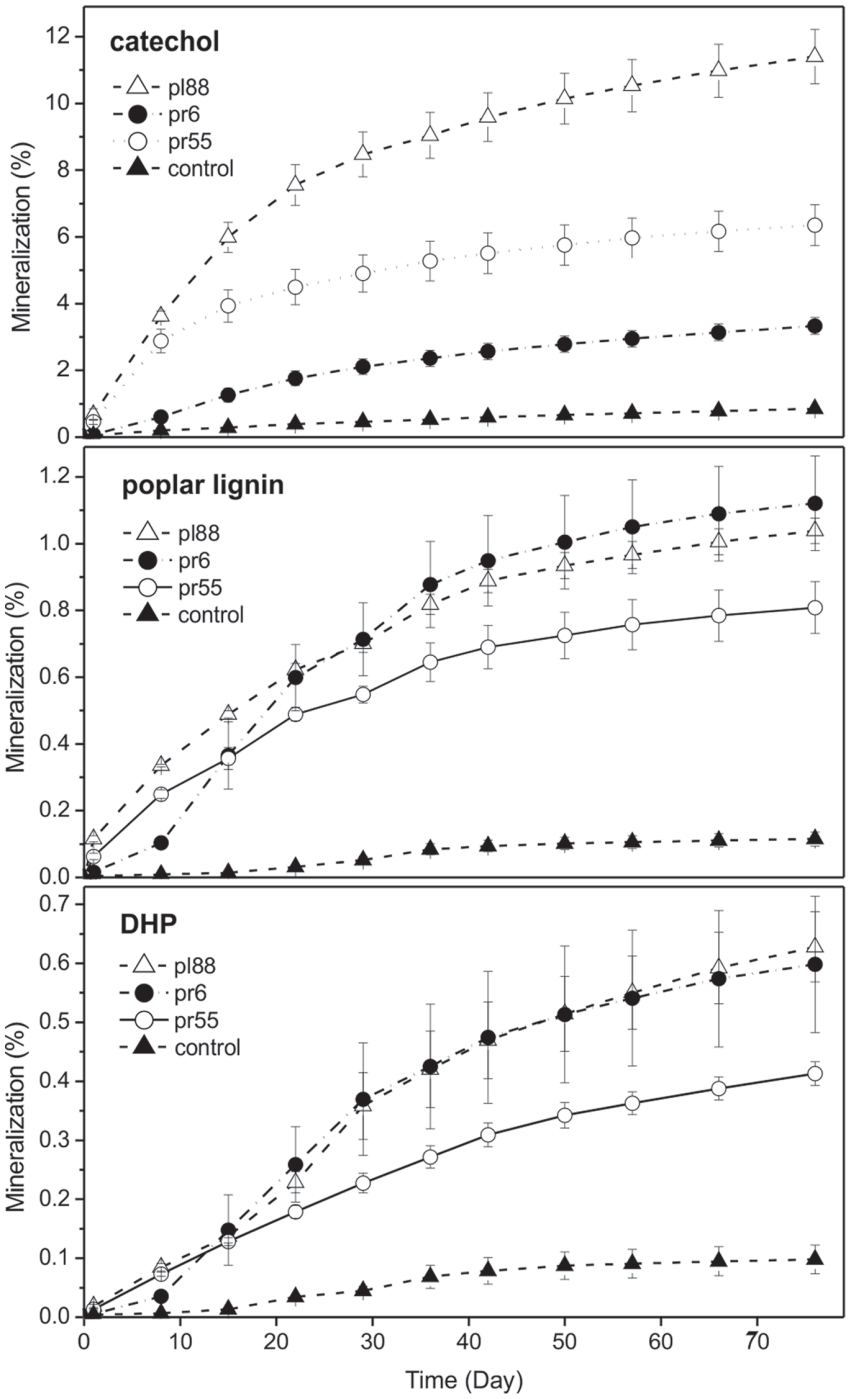
Mineralization of phenolic compounds by selected *Actinobacteria*. Time course of ^14^CO_2_ production during the transformation of ^14^C-catechol, ^14^C-poplar lignin and ^14^C-DHP in wheat straw microcosms by the selected actinobacterial strains. Control treatments contained sterile straw. The data represent the means and standard errors.

To assess whether bacteria that utilise polysaccharides are also able to cometabolise, that is, transform or even mineralise the phenolic compounds within lignocellulose, their transformation of ^14^C-labelled phenolic compounds was studied. Transformation was studied using compounds of various complexities that included the monomeric catechol (the precursor of lignin), the nonspecifically labelled poplar lignin that was isolated from plant tissues and ^14^C_β_-labelled DHP, which is a specifically labelled lignin model compound. After 76 days of cultivation of the bacteria on wheat straw supplemented with the ^14^C-labelled compounds, considerable mineralisation was only found for catechol: 11.4% for strain pl88, 6.4% for pr55 and 3.33% for pr6 (Figure 3). Mineralisation of poplar lignin was higher in pr6 and pl88 (>1%) than in pr55 (0.81%), and the same result was found for DHP, where mineralisation by pr55 was also lower than that of the other two strains. In all cases, inoculation with *Actinobacteria* resulted in significantly higher mineralisation than that of control flasks. The mass balance extraction of wheat straw showed that, in addition to the mineralisation of phenolic compounds, *Actinobacteria* increased the relative share of water-soluble phenolics, and this was most apparent for catechol, where insoluble ^14^C compounds represented 47% of the total in the control, while they were 19–26% of the control after incubation with bacteria. The amounts of soluble ^14^C in the DHP- and lignin-containing flasks also increased after bacterial treatment, but only slightly, by 2–4% and 0.7–2.0%, respectively (Table 3).

**Table 3:** Mass balance of ^14^C-catechol, ^14^C-poplar lignin and ^14^C-DHP lignin after a 76-day incubation in wheat straw microcosms with the selected actinobacterial strains.

Substrate	Strain	Respired		Soluble		Bound	
catechol	pl88	11.64±0.85	a	65.2±0.7	c	23.2±0.6	c
	pr6	3.33±0.29	b	70.6±1.1	b	26.1±0.8	b
	pr55	6.36±0.60	c	74.4±0.5	a	19.2±1.1	d
	control	0.94±0.02	d	52.2±0.1	d	46.8±0.1	a
poplar lignin	pl88	1.04±0.03	a	2.7±0.1	c	96.2±0.1	b
	pr6	1.12±0.13	a	4.0±0.2	a	94.9±0.2	c
	pr55	0.81±0.07	b	3.4±0.2	b	95.8±0.2	b
	control	0.09±0.04	c	2.0±0.2	d	97.9±0.3	a
DHP	pl88	0.67±0.06	a	21.7±0.4	a	77.7±0.4	b
	pr6	0.64±0.11	a	22.4±0.5	a	76.9±0.6	b
	pr55	0.45±0.02	b	23.1±0.5	a	76.4±0.6	b
	control	0.11±0.03	c	18.9±1.1	b	81.0±1.1	a

The data (% of the total) represent the means and standard errors. Different letters indicate statistically significant differences at *P*<0.05.

The mineralisation of lignin by *Streptomycetes* was first reported by Crawford, who found that mineralisation rates of ^14^C-lignin-labelled fir varied from 1.5 to 3% after nearly 42 days [57]. Similar rates were also reported by Pasti et al. [58]. Occasionally, higher lignin mineralisation rates were reported, such as 2.9% after 10 days by *Arthrobacter* sp. [59] or up to 5% after 15 days by *Nocardia* sp. [38]. In comparison, the mineralisation rates that were observed in our isolates seemed to be relatively low, but care must be taken in these comparisons because the modes of lignin labelling and preparation greatly affect mineralisation rates. The mineralisation rates of the recalcitrant lignin model compound ^14^C-DHP that were reported in this study indicates that the ability of the studied *Actinobacteria* to catalyse the complete decomposition of lignin is low and that cometabolic lignin degradation during the growth of active decomposers on lignocellulose is rather negligible.

Although solubilisation is the first important step in lignin decomposition because it makes the polar lignin residues available to other microorganisms, only a few studies have reported the amount of solubilised lignin. Our results indicate only a limited solubilisation of lignin, and these data are comparable to those published for several *Streptomycetes* (6–10%) [58].

Catechol is the first intermediate product of phenol degradation, and its cleavage is a critical step in the aerobic degradation of aromatic compounds in microorganisms [60]. Several bacterial strains that are capable of catechol degradation belong to various phyla, such as *Actinobacteria*, *Alphaproteobacteria*, *Betaproteobacteria* or *Gammaproteobacteria*. Catechol degradation was investigated mainly in studies that focused on the treatment of phenolic wastes, and efficient degraders were found in the genera *Pseudomonas*, *Acinetobacter* and *Klebsiella* [61]. All of the isolates in our study were able to efficiently degrade or solubilise catechol. Although the importance of phenolics as a growth substrate for bacteria is not clear, low molecular mass phenolic compounds are relatively common in litter [62] and their transformation might be of importance.

The results confirm the potential importance of *Actinobacteria* in lignocellulose degradation, although it is likely that the decomposition of biopolymers is limited to strains that represent only a minor portion of the entire community. Our results indicate that strains that are capable of growth on a complex lignocellulose substrate exist; these taxa are able to decompose cellulose and hemicelluloses, and at least some of them may prefer the latter. The importance of cometabolic degradation of lignin seems to be limited when compared with the abilities of saprotrophic fungi, but *Actinobacteria* may still contribute to the solubilisation of phenolics, especially low-molecular-mass compounds. Future studies using genomic, transcriptomic or proteomic approaches are needed to explore the link between the genetic potential of *Actinobacteria* and their actual activities as decomposers of organic matter.
